# Medical Works

**Published:** 1861-04

**Authors:** 


### A Work every Physician Wants.

### SUMMARY OF MEDICAL SCIENCES.

Comprising the essentially practical articles embraced in
BRAITHWAITE'S RETROSPECT, RANKING'S ABSTRACT,
THE LONDON, DUBLIN, EDINBURGH
MEDICAL JOURNALS.
FRENCH, GERMAN, AND OTHER
CONTINENTAL MEDICAL PERIODICALS.
commencing with 1860.

The whole in as concise a form as may be consistent with their respective merits in view of presenting to the Profession—

THE CREAM OF MEDICAL LITERATURE.

In additton to such resume will be embodied contributions from
EMINENT AMERICAN PHYSICIANS AND SUGEONS.

**Edited by WALTER S. WELLS, M. D.,**
Compiler of the Epitome of Braithwaite's Retrospect.

The first part of the SUMMARY OF MEDICAL SCIENCES will be ready April 1st, and will be published semi-annually. Each part will contain about 300 octavo pages of closely printed matter—uniform with "THE EPITOME"—and will be classified and paged for the convenience of binding under the respective headings of PRACTICAL MEDICINE, SURGERY, MIDWIFERY, CHEMISTRY AND TOXICOLOGY, AND MATERIA MEDICA.

**Terms of Publication:**

The Summary will be published and delivered, free of expense, to any part of the United States (within 3,000 miles) at $2.00 per annum, in advance.

Single parts, $1.25.

CHARLES T. EVANS, Publisher,
532 Broadway (up stairs), New York.

☞ Physicians desirous of examining Part one will be supplied with a copy by sending $1.00 to the Publisher.

*** All remittances must be in New York Funds. The Publisher will be responsible for all sums remitted in registered letters.

The Summary will be sent to all Medical Journals who will editorially announce the Publication. Send copies to the Publisher.

*NOW READY,*

### Epitome of Braithwwaite's Retrospect of Practical Medicine & Surgery

BY W. S WELLS, M. D.

2 vols, large 8vo. (900 pages each), sheep $7.00.

"*You have rendered an* ESSENTIAL SERVICE TO THE PROFESSION *by your* 'EPITOME' *of the extensive* 'RETROSPECT OF BRAITHWAITE.'"—VALENTINE MOTT, M. D.

*From the North American Medico-Chirurgical Review.*

"This Epitome, which has been prepared with great care, should be in the library of every phyrician in the country."

*From the Atlanta Medical and Surgical Journal.*

"We regard this as one of the most valuable contributions of the day, and as a speeial favor conferred upon the profession."

The above are only a few of the notices of the work which the publisher has received. Without an exception the Medical Press has spoken of it as a NECESSITY to the Profession. Subscribers to the work who have already purchased it speak of it in the sameflattering manner.

Published by C. T. EVANS, 532 Broadway, New York.

☞ On receipt of the price in Funds current at New York, the copies of work will be sent to any part of the United States (within 3,000 miles) free of expense.

*** All remittances, if sent in registered letters, are at the risk of Publisher.

